# Cytokines in Acute Chikungunya

**DOI:** 10.1371/journal.pone.0111305

**Published:** 2014-10-24

**Authors:** Anuradha Venugopalan, Ravi P. Ghorpade, Arvind Chopra

**Affiliations:** Center for Rheumatic Diseases, Pune, India; Mount Sinai School of Medicine, United States of America

## Abstract

**Introduction:**

Acute chikungunya (CHIKV) is predominantly an acute onset of excruciatingly painful, self-limiting musculoskeletal (MSK) arbovirus illness and this was further reported by us during the 2006 Indian epidemic [Chopra et al. Epidemiol Infect 2012]. Selected serum cytokines profile in subjects within one month of onset of illness is being presented.

**Methods:**

Out of 509 clinical CHIKV cases (43% population) identified during a rural population survey, 225 subjects consented blood investigations. 132 examined within 30 days of febrile onset are the study cohort. Anti-CHIKV IgM and IgG antibodies tested by immunochromatography and indirect immunofluorescence respectively. Interferons (IFN)-α, -β and -γ, Interferon Gamma-Induced Protein-10 (CXCL-10/IP-10), Tumor Necrosis Factor-α (TNF-α), Interleukin-1β (IL-1β), Interleukin-6 (IL-6), Interleukin-13 (IL-13), Monocyte Chemoattractant Protein-1 (MCP-1), Interleukin–4 (IL-4) and Interleukin–10 (IL-10) performed by ELISA. Samples collected from neighboring community a year prior to the epidemic used as healthy controls.

**Results:**

Seropositivity for anti-CHIKV IgM and IgG was 65% and 52% respectively. IFN-α, IFN-β, IFN-γ, CXCL10/IP-10 and IL-1β showed intense response in early acute phase. Cytokines (particularly TNF-α, MCP-1, IL-4, IL-6 and IL-10) was maximum in extended symptomatic phase and remained elevated in recovered subjects. Higher (p<0.05) IFN and IL-4 seen in patients seropositive for anti-CHIKV IgG. Elderly cases (≥65 years) showed elevated cytokines (except IFN) and anti-CHIKV antibodies near similar to younger subjects. Significant correlations (p<0.05) found between cytokines and clinical features (fatigue, low back ache, myalgia) and anti-CHIKV antibodies.

**Conclusion:**

An intense cytokine milieu was evident in the early and immediate persistent symptomatic phase and in recovered subjects. Early persistent IgM and lower IgG to anti-CHKV and intense Th2 cytokine phenotype seem to be associated with delay in resolution of MSK symptoms. Intriguingly, maximum TNF-α, IL-6 and IL-13 with low anti-CHIKV IgM response found in subjects recovered from CHIKV within one month of illness.

## Introduction

Chikungunya (CHIKV), an arbovirus belonging to the family *Togaviridae*, is transmitted by mosquito (*Aedes aegypti* and *A. albopictus*) and causes an acute onset of febrile excruciatingly painful musculoskeletal (MSK) illness which is predominantly selflimiting. A reemergence of an African strain of the CHIKV in 2006 caused a widespread unprecedented epidemic in South Asia and several neighboring regions and islands [1]–[7]. Inundated by several hundred clinical referrals to the rheumatology outpatient, we reported a unique spectrum of arthritis and rheumatism in naïve patients following the CHIKV illness [8]. We carried out a rural community survey during the epidemic and reported the clinical profile of acute CHIKV and its MSK sequel [1]. We were intrigued by the sheer intensity of musculoskeletal pain (MSK) and polyarthralgias at the onset of illness but the large majority of our cases recovered within three weeks. However, about one third of cases continued to suffer from persistent MSK pain and arthritis beyond one month of onset of illness. This was much more than what was reported from earlier epidemics [9]. To explore this further, we carried out a detail laboratory evaluation of the rural community survey subjects which included selected cytokine assay. The purpose was to unravel some of the immune pathological correlations and patterns of cytokine upregulation. In this report, we primarily focus on the cytokine profile of the cases which were examined within four weeks of the onset of CHIKV illness.

## Methods

The protocol was approved by the ethic committee of CRD, Pune. All eligible patients were provided a study information brochure and signed an informed consent form (local language) prior to enrollment.

The principal study [1], [2] was designed as a house to house rural population survey during the Indian epidemic of CHIKV in 2006 in Bavi (a village in district Sholapur, state of Maharashtra, West India). The currentstudy design used was a cross sectional analysis of cytokine assay in subjects diagnosed with CHIKV and evaluated within four weeks (current study period) of the onset of febrile illness.

### Definition and Classifications

For the study an epidemic based definition, decided a-priori by the rheumatologist (AC), was used to diagnose CHIKV. It was decided that, an acute onset of febrile painful typical MSK illness in a naïve subject during the epidemic period (with several communities suffering from similar illness) was sufficient for the clinical diagnosis. However, the subjects under the study were carefully screened for common community infections (in particular malaria and dengue) and subjected to serological testing of anti CHIKV antibodies. All cases (survey) were clinically examined and empirically classified as CHIKV by AC and other team doctors, as per protocol. Typical cases were characterized by a short duration (<3 days) of high fever accompanied by severe debilitating polyarthralgias and musculoskeletal aches and pains lasting less than 10 days. Subjects with milder intensity of CHIKV like illness (and/or other symptoms like fatigue, headaches, skin lesions) and lasting up to seven days were labeled ‘probable’ and were observed further. All individuals who were asymptomatic but provided a typical prior history of acute CHIKV illness suffered during the epidemic were included if the first evaluation (clinical and laboratory) was carried out within the study window period (four weeks) and labeled ‘recovered’. All cases which tested seropositive for anti CHIKV antibody (IgM and/or IgG) were classified as ‘definite cases’.

Based on the time point of the first clinical examination (blood collected simultaneously) following the onset of the illness (fever), cases were empirically classified into acute (0–5 days), subacute (6–14 days) and extended phase (15–30 days) illness.

### Blood Samples

Blood samples were aseptically collected and suitably transported (road) in ice packs to the Center for Rheumatic Diseases: Research & Diagnostic (CRD: R & D) Lab, Pune within 6–8 hours of collection. Serum was separated immediately and stored at −80°C. Pune city was 156 kms from the Bavi survey site. Routine hematology and immunology tests were carried out within 24 hours of blood collection. Peripheral blood smears were examined to exclude malarial parasite. Acute phase reactants included erythrocyte sedimentation rate (ESR, Westergren, reported as mm fall at end of 1^st^ hour) and C - reactive protein (CRP, using nephelometry; cut off value 5 mg/L). The autoantibody profile included Rheumatoid Factor (RF, estimated by nephelometry; cut off value 40 IU/ml), anti-Cyclic Citrullinated Peptide (anti CCP, commercially available second generation ELISA, Euroimmun, Germany; cut off value of ≥5 RU/ml) and Antinuclear Antibody (ANA, using qualitative enzyme immunoassay).

### Anti CHIKV Antibodies

Specific IgM and IgG antibodies to CHIKV were tested by immunochromatography [10] and indirect immunofluorescence respectively. Due to limited budget grant, it was decided to test for anti CHIKV antibody IgG in all the acute phase subjects and those blood samples available at more than two follow-up visits. In the current study cohort, IgG was tested in 58 subjects [42 symptomatic (acute phase = 23, subacute phase = 3, extended symptomatic phase = 16) and 16 recovered subjects]. Anti-dengue antibodies (IgG and IgM) were also tested in all samples using On-site lateral flow chromatographic immunoassay.

### Serum Cytokines

In the study, Interferon (IFN) –α, -β and -γ, Tumor Necrosis Factor - α (TNF-α), Interleukin (IL) −1β, IL-6, IL-13, Interferon Gamma-Induced Protein-10 (CXCL-10/IP-10), Monocyte Chemoattractant Protein-1 (MCP-1), IL –4 and IL-10 were measured using development kits (R&D Systems, USA). The DuoSeT ELISA Development kit (R&D Systems Inc., USA) contained the basic components required for the development of sandwich ELISAs to measure natural and recombinant cytokines. The assay procedure was similar for all cytokines. The working concentrations of capture antibody, detection antibody and standards for each of the individual components was as per the literature provided by the manufacturer. Due to limited study grant budget IFN-α, IFN-β, IL-1β, MCP-1, IL-4 and IL-10 were measured 31 in symptomatic cases (acute phase = 23; subacute phase = 4; extended symptomatic phase = 4) and in 80 healthy controls.

Based on standard literature, cytokines were broadly classified into Th1 (IFN –α, -β and -γ, TNF-α, IL- 1β, CXCL-10/IP-10, MCP-1) and Th2 (IL-6, IL –4, IL-10, IL-13) phenotype.

### Study Cohort

**Figure 1:**
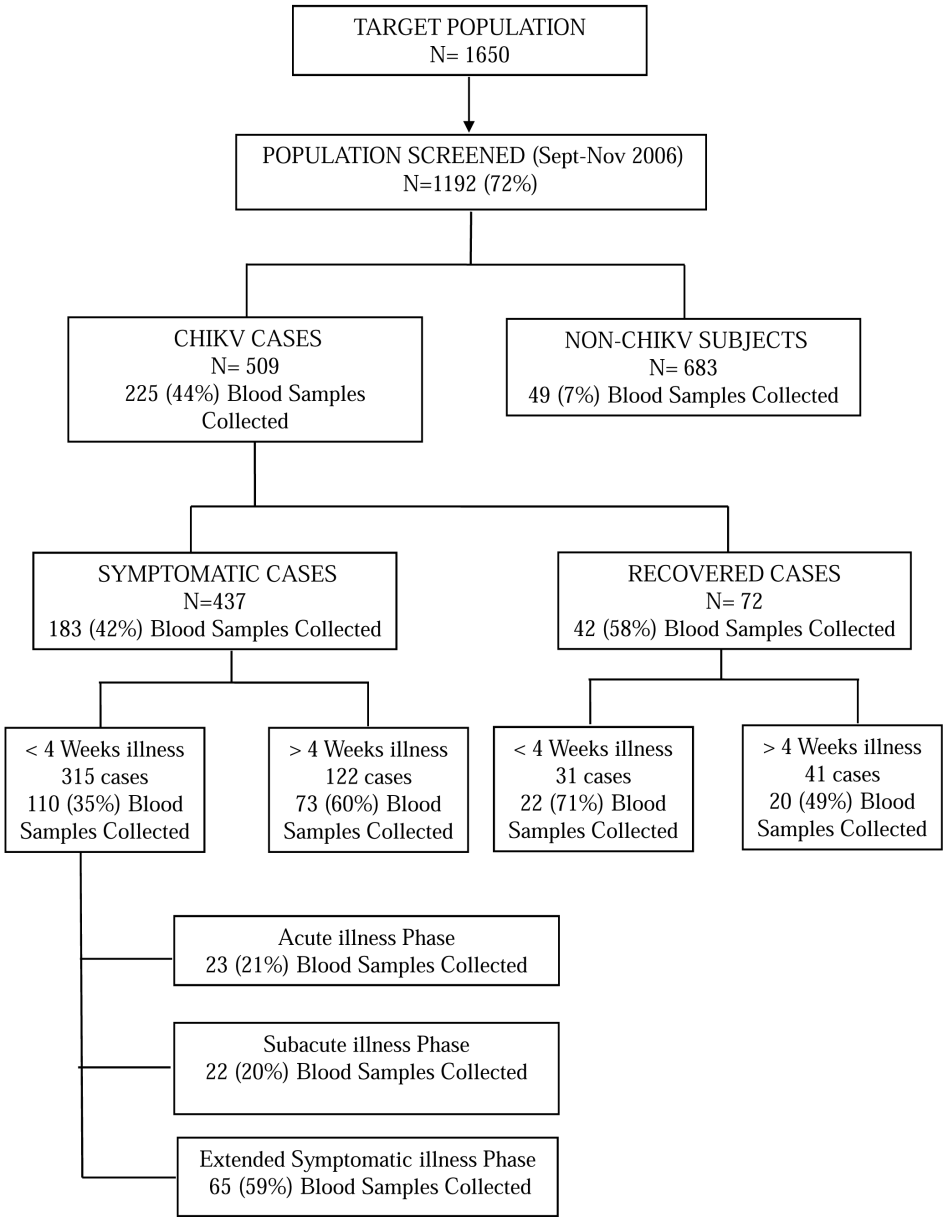
Number of cases identified and blood samples collected during survey and at subsequent follow-up period.

The current study cohort for cytokine assay was made up of 132 cases (110 symptomatic and 22 recovered) who were first evaluated (clinical and laboratory) during the epidemic survey within 4 weeks of onset of fever (Fig. 1) The male:female ratio in the symptomatic and recovered groups was 1∶1.5 and 1∶3 respectively. The median age was 36 years in both groups.

The study cohort was drawn from 225 community cases (44% survey cases) who consented to donate blood for laboratory investigations and a selection was made on first come first serve basis (Fig. 1).

Several cases were evaluated at regular intervals and followed up for periods ranging from 6–22 months as per the survey protocol. The follow up data is not included in the current study report.

### Survey Controls

49 subjects in the village who did not recall having had fever or any symptoms suggestive of CHIKV infection (during the CHIKV epidemic) also consented to give blood samples during the survey.

### Healthy Controls

Coincidentally, Bavi village was close to our rural project site (Bhigwan) for an ongoing WHO ILAR (International League of Associations for Rheumatology) COPCORD (Community Oriented Program for Control of Rheumatic Diseases) since 1996 [11]. Blood samples (N = 80) collected from consenting healthy villagers (without any symptomatic chronic illness and in particular arthritis) examined during a COPCORD activity in 2005 (a year prior to the CHIKV epidemic of 2006) and stored at −80°C in CRD lab were used as healthy controls. The male : female ratio was 1∶1 and the median age was 41 years. These samples tested negative for dengue and CHIKV antibodies.

### Statistics

The survey sample size was not based on any statistical (power of the study) calculation. SPSS version 12.0 (USA) was used for all statistical calculations. The differences in serum cytokines between the groups was compared by a non-parametric analysis with Kruskal – Wallis test with significance at p<0.05. Standard Pearson’s correlation coefficient was calculated and the strength (correlation) was classified as weak (0.1–0.3), moderate (0.4–0.6), strong (0.7–0.9) and perfect (1.0). Linear regression analysis was performed to determine predictors of resolution of symptoms within one month in the study cohort.

## Results

Fig. 1 provides an overview of the survey subjects screened, cases detected, cases with blood sample collection and distribution of various subject groups characterized by symptomatic status and illness duration at the time of initial evaluation. As per the study protocol, all cases in the current study cohort were clinically classifiable as typical or definite (see above).

### 1 Anti CHIKV antibodies

The serological profile of the study cohort is shown in Table 1. 51% symptomatic and 32% recovered subjects tested seropositive for specific anti-CHIKV IgM antibodies; corresponding positivity for anti-CHIKV IgG antibody was in 40% symptomatic and 50% recovered subjects. 4% and 8% survey controls (without history of CHIKV) tested positive for anti-CHIKV IgM and IgG antibody respectively. The proportion of cases with seropositive IgM was observed to increase with higher duration of illness; 17% acute, 55% sub acute and 62% extended symptoms. In contrast, the proportion of cases with seropositive IgG was much lesser with increased duration; 52% acute, 100% sub acute and 13% extended symptoms. 32% and 50% of recovered cases in the study cohort were seropositive for anti CHIKV IgM and IgG antibody respectively.

**Table 1:** Serological profile (frequency of positive result) of symptomatic cases (N = 110) with recovered cases (N = 22) of CHIKV with illness within one month duration.

Test	Acute (N = 23)*	Subacute (N = 22)*	Extended Symptomatic (N = 65)*	Total Symptomatic (N = 110)*	Recovered (N = 22)*
	N (%)	N (%)	N (%)	N (%)	N (%)
CHIKV IgM +	4 (17%)	12 (55%)	40 (62%)	56 (51%)	7 (32%)
CHIKV IgG+	12 (52%)	3 (100%)	2 (13%)	17 (40%)	8 (50%)
CHIKV IgM+IgG−	1 (4%)	0	10 (63%)	11 (26%)	2 (13%)
CHIKV IgM−IgG+	9 (39%)	1 (33%)	0	10 (24%)	4 (25%)
CHIKV IgM+IgG+	3 (13%)	2 (67%)	2 (13%)	7 (17%)	4 (25%)
CHIKV IgM−IgG−	10 (43%)	0	4 (25%)	14 (33%)	6 (38%)
CRP (≥6 mg/L)	6 (26.1%)	2 (9.1%)	8 (12.3%)	16 (14.5%)	4 (18.2%)
RF (≥40 IU/ml)	2 (8.7%)	0	3 (4.6%)	5 (4.5%)	0
ANA	1 (4.3%)	1 (4.5%)	3 (4.6%)	5 (4.5%)	2 (9.1%)

CRP = C-reactive Protein; RF = Rheumatoid Factor; ANA = Antinuclear Antibody.

*Note : Anti CHIKV IgM was tested in all the 110 symptomatic and 22 recovered subjects; Anti CHIKV IgG was only tested in 42 symptomatic subjects (Acute = 23; subacute = 3 and extended symptomatic = 16) and 16 recovered subjects; CRP, RF and ANA was tested in all subjects.

### 2 Acute Phase Reactants

Though not strictly fasting, the mean ESR was 75 mm fall at the end of 1^st^ hour (range 5–89). 16 (14.5%) cases in the study cohort were seropositive for CRP with a median value of 12 mg/L (range 6–48). A higher proportion of cases (26.1%) showed seropositive CRP in the acute phase illness as compared to subacute and extended duration (Table 1). 18.2% recovered cases were seropositive for CRP.

### 3 Serum Cytokines

**Figure 2:**
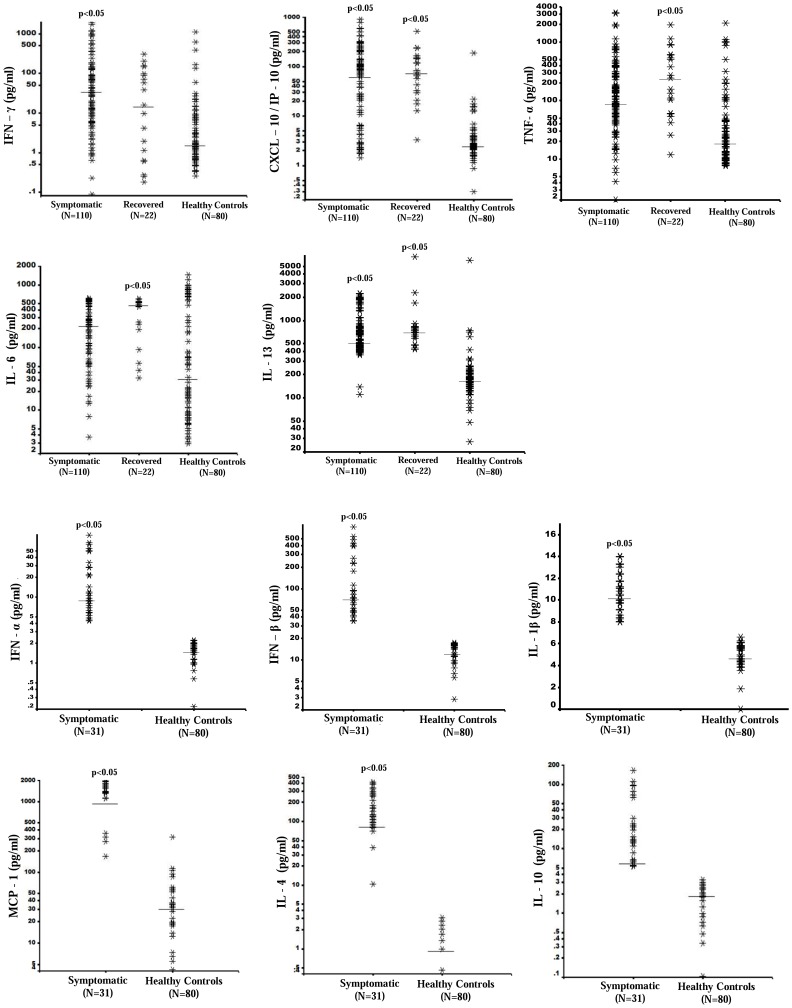
Selected Serum cytokine profile at of CHIKV cases (Symptomatic N = 110; Recovered N = 22) with CHIKV illness duration of 30 days compared with Healthy Controls (N = 80). (*Horizontal bars indicate medians of each category.).* **Footnote to** **Figure 2** **:** IFN-Interferon; TNF = Tumor Necrosis Factor; CXCL-10/IP-10 = Interferon Gamma-Induced Protein-10; IL = Interleukin; MCP = Monocyte Chemoattractant Protein. Statistical analyses were performed using non-parametric Kruskal Wallis test. ‘p’ values indicate significant difference between the corresponding group when compared with healthy control group. No significant (p<0.05) differences were observed between the symptomatic (N = 110) and recovered groups (N = 22) for any of the cytokines.

Fig. 2 shows the scatter distribution of various cytokines assay in the study cohort and healthy controls. Interestingly, several healthy control cases showed strikingly high assay of IFN-γ, TNF-α and IL-6. However, the serum cytokines assay (median) was significantly higher (p<0.05) in the study cohort (symptomatic and recovered) as compared to that observed in the healthy controls (Fig. 2). Except for IFN-γ, the serum assays (median) for TNF-α, IL-6, IL-13, CXCL-10/IP-10, were higher in the recovered group (Fig. 2) as compared to healthy control [IL-6 (p = 0.01), IL-13 (p = 0.06)]; assay for other cytokines could not be carried out in this group.

Table 2 shows serum cytokine assay in the acute, subacute and extended symptomatic sub-groups. Compared to healthy controls (Fig. 2), all cytokines were elevated several folds in each of the subgroups. In the symptomatic group, maximum elevation for IFN (α, β, γ), CXCL-10/IP-10, IL-1β and IL-13 was observed in the acute phase illness and TNF-α, MCP-1, IL-6 and IL-10 in patients with extended symptoms. It was observed that IL-4 (marker for Th2 cytokine response) was maximal in the subacute illness group (7–14 days post onset). However, on comparison across board, IL-4, TNF-α, CXCL-10/IP-10, IL-6 and IL-13 was maximally elevated in the recovered group.

**Table 2:** Cytokine profile of cases with CHIKV illness within one month duration (N = 110).

Cytokine	Acute Phase	Subacute Phase	Extended Symptomatic Phase	Total Symptomatic	Recovered	Healthy Controls
(pg/ml)	n = 23	n = 22^#^	n = 65^#^	n = 110	n = 22	n = 80
	14.16	5.71	4.68	9.61		1.58
IFN-α	[23.51]	[0.26]	[0.40]	[22.32]	ND	[0.50]
	(8.90–4.90)	(5.37–5.82)	(4.40–5.16)	(5.84–28.42)		(1.15–1.90)
	112.57	46.24	38.11	76.00		13.36
IFN-β	[191.88]	[2.57]	[3.43]	[181.64]	ND	[3.92]
	(70.10–390.40)	(43.11–47.82)	(35.28–41.70)	(48.26–227.99)		(9.82–16.25)
	85.24	29.33	26.84	37.70	15.82	1.72
IFN-γ	[476.59]	[201.56]	[329.49]	[348.42]	[86.18]	[153.13]
	(30.61–416.17)	(10.02–76.40)	(8.34–127.36)	(9.62–133.69)	(0.64–97.57)	(0.88–8.22)
	71.44	61.81	66.15	65.65	79.48	2.65
CXCL-10/IP-10	[277.41]	[118.57]	[93.85]	[161.87]	[111.97]	[21.44]
	(21.76–415.95)	(6.09–97.82)	(5.98–99.86)	(6.67–107.23)	(31.79–149.04)	(2.06–3.96)
	11.15	8.56	8.19	10.44		4.92
IL-1β	[1.49]	[0.32]	[0.18]	[1.82]	ND	[1.34]
	(10.02–12.44)	(8.41–8.99)	(8.02–8.36)	(9.11–11.80)		(4.19–5.70)
	90.99	94.30	107.54	97.20	257.88	20.65
TNF-α	[26.12]	[60.38]	[231.76]	[355.80]	[475.71]	[328.60]
	(18.01–188.33)	(4.2–1959.1)	(57.79–276.20)	(53.05–229.05)	(60.69–610.03)	(11.73–50.51)
	1576.40	1218.88	1869.23	1605.09		32.23
MCP-1	[541.83]	[584.95]	[776.26]	[570.59]	ND	[76.45]
	(1333.64–1892.25)	(514.56–1608.27)	(711.79–1955.56)	(1254.59–1892.25)		(18.31–58.73)
	119.58	392.57	170.53	137.94		1.00
IL-4	[89.36]	[34.10]	[24.26]	[111.85]	ND	[0.61]
	(88.98–249.31)	(347.36–409.73)	(162.00–204.77)	(96.83–273.78)		(1.00–1.69)
	172.39	233.74	250.93	235.07	501.16	33.40
IL-6	[230.68]	[142.59]	[197.89]	[194.47]	[203.08]	[354.81]
	(29.19–513.40)	(103.16–278.85)	(86.60–498.81)	(82.21–456.99)	(224.57–557.53)	(8.46–472.96)
	13.12	46.12	72.97	17.44		1.98
IL-10	[43.88]	[22.11]	[11.87]	[41.47]	ND	[0.87]
	(6.46–22.05)	(25.84–66.39)	(68.89–89.82)	(8.17–68.90)		(1.91–2.55)
	581.34	449.69	576.09	558.63	751.61	177.77
IL-13	[46.63]	[345.68]	[939.43]	[784.39]	[1315.41]	[688.76]
	(420.15–780.05)	(427.82–830.07)	(461.38–1093.22)	(440.37–856.74)	(606.44–835.81)	(142.29–212.07)

*Values expressed as median [standard deviation](interquartile range) pg/ml.*

IFN-Interferon; TNF = Tumor Necrosis Factor; CXCL-10/IP-10 = Interferon Gamma-Induced Protein 10; IL = Interleukin; MCP = Monocyte Chemoattractant Protein. **^#^Note** : IFN-α, IFN-β, IL-1β, MCP-1, IL-4 and IL-10 were tested in 4 subjects each of subacute phase and extended symptomatic phase. All other cytokines were tested in 23, 22 and 65 subjects of acute, subacute and extended symptomatic phase respectively. Statistical analyses were performed using non-parametric Kruskal Wallis test for significant differences within subgroups in the symptomatic group and for differences within total symptomatic group and recovered group versus healthy controls.

### 4 Serum Cytokine Correlations

Table 3 and Table 4 show significant (p<0.05) correlations (positive and negative) between cytokines and selected clinical and laboratory features and specific anti CHIKV antibodies.

**Table 3:** Significant positive correlations between cytokines and selected clinical and laboratory variables.

Cytokine	Acute Phase	Subacute Phase	Extended Symptomatic Phase	Recovered	Total
(pg/ml)	n = 23	n = 22	n = 65	n = 22	N = 132
	IFN-β (1.00)				IFN-β (1.00)
IFN-α	IL-1β (0.94)	ND	ND	ND	IL-1β (0.90)
	CXCL-10/IP-10 (0.59)				CXCL-10/IP-10 (0.51)
IFN-β	CXCL-10/IP-10 (0.50)	ND	ND	ND	CXCL-10/IP-10 (0.52)
					Fatigue (0.42)
					Pain Intensity (0.23)
	CXCL-10/IP-10 (0.45)	–	Platelet Count (0.35)	Low Backache (0.48)	CXCL-10/IP-10 (0.18)
IFN-γ	MCP-1 (0.65)		CXCL-10/IP-10 (0.25)	TNF-α (0.51	MCP-1 (0.74)
					IL-6 (0.31)
					IL-13 (0.18)
		CHIKV-G (0.83)			TNF-α (0.24)
CXCL-10/IP-10	IL-6 (0.57)	Platelet Count (0.52)	CHIKV-G (0.58)	–	IL-10 (0.37)
		IL-6 (0.62)			IL-6 (0.39)
					IL-13 (0.45)
					Fatigue (0.47)
IL-1β	CXCL-10/IP-10 (0.61)	ND	ND	ND	CRP (0.37)
					CXCL-10/IP-10 (0.41)
					Headache (0.20)
TNF-α	IL-6 (0.48)	Headache (0.48)	IL-6 (0.60)	Hemoglobin (0.44)	IL-6 (0.61)
					IL-13 (0.23)
					Platelet Count (0.41)
MCP-1	–	ND	ND	ND	TLC (0.37)
					♀ Gender (0.38)
IL-4	CHIKV-M (0.58)	ND	ND	ND	Myalgia (0.47)
					IL-10 (0.85)
IL-6	CRP (0.54)	CHIKV-G (0.72)	CHIKV-G (0.53)	–	Skin Rash (0.19)
	♀ Gender (0.48)	TLC (0.51)			IL-13 (0.26)
IL-13	–	Headache (0.52)	CRP (0.28)	–	–

(*Pearson correlation co-efficient in parenthesis.*).

IFN-Interferon; TNF = Tumor Necrosis Factor; CXCL-10/IP-10 = Interferon Gamma-Induced Protein-10; IL = Interleukin; MCP = Monocyte Chemoattractant Protein. ND = Not Done; TLC = Total Leucocyte Count; CRP = C-reactive protein; ♀ = Female.

**Table 4:** Significant negative correlations between cytokines and selected clinical and laboratory variables.

Cytokine	Acute Phase	Subacute Phase	Extended Symptomatic Phase	Recovered	Total
(pg/ml)	n = 23	n = 22	n = 65	n = 22	N = 132
IFN-α	IL-4 (−0.73)	ND	ND	ND	CHIKV-M (−0.51)
	IL-10 (−0.44)				Myalgia (−0.37)
					IL-4 (−0.69)
					IL-10 (−0.51)
IFN-β	IL-4 (−0.73)	ND	ND	ND	Myalgia (−0.37)
	IL-10 (−0.44)				CHIKV-M (−0.50)
					IL-4 (−0.68)
					IL-10 (−0.51)
IFN-γ	–	–	Headache (−0.27)	–	–
					
CXCL-10/IP-10	TLC (−0.51)	–	–	–	–
	MCP-1 (−0.54)				
	IL-4 (−0.52)				
IL-1β	IL-4 (−0.86)	ND	ND	ND	CHIKV-M (−0.69)
	IL-10 (−0.56)				IL-4 (−0.77)
	CHIKV-G (−0.49)				IL-10 (−0.62)
TNF-α	Hemoglobin (−0.47)	TLC (−0.56)	–	Age ≥45 years (−0.49)	–
	Myalgia (−0.48)				
MCP-1	Platelet Count (−0.54)	–	–	–	–
	♀ Gender (−0.51)				
	IL-6 (−0.45)				
IL-4	IL-6 (−0.42)	ND	ND	ND	–
					
IL-10	IL-6 (−0.45)	ND	ND	ND	–

(*Pearson correlation co-efficient in parenthesis*.).

IFN-Interferon; TNF = Tumor Necrosis Factor; CXCL-10/IP-10 = Interferon Gamma-Induced Protein-10; IL = Interleukin; MCP = Monocyte Chemoattractant Protein. ND = Not Done; TLC = Total Leucocyte Count; CRP = C-reactive protein; ♀ = Female.

#### 4.1 Acute phase

Moderate to perfect positive correlations were found between several cytokines. Each of the interferon subtypes showed moderate positive correlation with chemokine CXL-10/IP-10. IL-4 showed moderate positive correlation with anti CHIKV IgM. A moderate negative correlation was seen between IL-1β and anti CHIKV IgG antibody. As is well known, a significant moderate positive correlation was demonstrated between IL-6 and CRP, and TNF-α and IL-6. A moderate positive correlation was also observed between myalgias and the pro-inflammatory cytokine TNF-α. Several pro-inflammatory cytokines (CXCL-10/IP-10, MCP-1 and TNF-α) individually showed moderate negative correlation with blood hemoglobin and cell counts (leucocytes, platelets). Inflammation leads to increased iron utilization and TNF-α and pro-inflammatory cytokines contribute to chronic anemia [12]. Significant moderate to strong negative correlations was observed between several Th1 cytokines (IF and CXCL-10/IP-10 ) and Th2 cytokines (MCP-1, IL-10 and IL-6) suggesting counter check immune inflammation mechanisms.

#### 4.2 Subacute phase

There were several interesting departures from the correlation pattern seen in the acute phase. In contrast, leukocyte and platelet counts gained positive correlations (CXCL-10/IP-10 and IL-6). TNF-α and IL-13 showed moderate positive correlation with headache. A strong correlations was observed for serum anti CHIKV IgG response and some cytokines (CXCL-10/IP-10 and IL-6).

#### 4.3 Extended symptomatic phase

Several moderate to strong correlations seen with the cytokines in the sub acute illness were observed in this group especially with reference to anti CHIKV IgG antibody response. The correlation between TNF-α and IL-6 was further strengthened (0.60). However, the overall correlation pattern appeared much different from that seen in the acute phase and in particular with loss of several negative correlations (Table 3 and Table 4).

#### 4.4 Recovered group

In the recovered group, IFN-γ correlated positively with a history of low backache and with TNF-α. TNF-α showed a moderate positive correlation with blood hemoglobin and we suspect that the improved hemoglobin level is due to recovery from illness. As stated earlier, for reasons not well understood several cytokines including TNF-α persisted high in the recovered group (Table 2). A moderate negative correlation was also seen between blood hemoglobin and age.

#### 4.5 Study cohort (symptomatic and recovered)

Several of the moderate, strong positive and negative correlations observed in the acute phase remained more or less unchanged when the total study cohort was considered. Some positive clinical correlations (weak to moderate) of interest need to be mentioned- IFN-γ and pain intensity, IFN-β, IL-1β individually and fatigue, TNF-α and headache, IL-4 and myalgia, IL-6 and skin rash. A moderately negative correlation was seen between IFN-α and myalgias. A moderate correlation between MCP-1 and leukocyte and platelet count was only seen in the study cohort. In contrast to positive correlations observed in the subgroups, significant moderate negative correlations were observed between anti CHIKV IgM antibody response and each of three cytokines individually - IFN- α, IFN-β and IL-1β. Both IL-6 and IL-13, showed significant but weak positive correlation with several Th1 cytokines (IFN -γ, CXCL-10/IP-10, and TNF-α).

### 5 Cytokine Responses and Anti-CHIKV Antibody Seropositivity

Cytokine responses were compared between the two groups - seropositive and seronegative anti-CHIKV IgM and IgG antibodies (Table S1, Table S2, Table S3, Table S4, Table S5, Table S6, Table S7 and Table S8 in File S1).

#### 5.1 Acute phase

An intense IFN-α/−β, IL1β, CXCL-10/IP-10, TNF-α and IL-6 response was seen in the seronegative group (often p<0.05) as compared to seropositive groups and may suggest a blunted Th1 response once the specific antibodies appear. (Table S1, Table S2 and Table S3 in File S1). Significantly elevated (p<0.05) IL-4 and IL-10 in the seropositive group suggested B cell activation.

#### 5.2 Subacute phase

The results were similar to those observed in acute phase except that the assays though elevated were comparatively reduced (Table S4 in File S1). There was no significant difference in IL-4 and IL-10 between the two groups and this may be attributed to a non-selected cytokine response once a robust IgM response is obtained.

#### 5.3 Extended symptomatic phase

Overall the observations were similar except for a further reduction in cytokine assays in both groups (Table S5 in File S1). There was an increase in IL-10 in the seropositive IgM group as compared to acute and subacute illness. IL-10 is a potent inhibitor of Th1 cytokine response and in this study it may reflect a host attempt towards recovery from illness.

#### 5.4 Recovered group

In the recovered group, there were no differences between seropositive and seronegative IgM groups for cytokine assay except for IFN-γ (increased in seronegative IgM group) (Table S6 in File S1).

#### 5.5 Study cohort (Antibody response

Overall the results were similar to those obtained in individual subset groups (Table S1, Table S4 and Table S5 in File S1) with respect to anti CHIKV IgM response in the symptomatic group (Table S7 in File S1); significantly elevated IL-4 and IL-10 was observed with seropositive IgM. When IgM positive cases were compared with IgG positive cases in the symptomatic group, IFN-α, IFN-β and IL-1β responses was significantly higher among the IgG positive group (Table S8 in File S1). Though not significant (p = 0.07), a much higher IL-6 response in the IgM group (Table S8 in File S1) as compared to IgG may suggest an ongoing intense inflammation with a failure to mount (switch) an IgG response. The persistent early elevation of MCP-1 and IL-13 observed in both, seropositive and seronegative groups of subjects with varying illness duration and recovered individuals does not support any role for these cytokines in antibody production.

Finally, the cytokine repertoire was compared between subjects of seropositive and seronegative groups for specific anti CHIKV antibodies (IgM/IgG) (Table S10 in File S1). Consistent with earlier observations (Table S1, Table S2 and Table S8 in File S1), IFN-α, -β and IL-10 were significantly elevated in seronegative group while IL-4 and IL-10 were significantly elevated in seropositive group. Figure S1 shows the scatter plot for cytokines in cases with serologically confirmed diagnosis of CHIKV.

### 6 Regressions

We further performed a step-wise linear regression using several clinical and lab variables in order to determine predictors of ‘failure to remission of musculoskeletal symptoms within one month of onset of illness’. Anti CHIKV IgG antibody was not included among the independent variables. IL-6, CXCL-10/IP-10, CRP and presence of knee pain and myalgia were found to significantly predict persistence of post CHIKV musculoskeletal pain.

### 7 Age and Response

The cytokine and anti CHIKV antibody response was classified as per age groups (Table S9 in File S1). We observed that 69% in the age group of less than 20 years, 53% in age group 21–44 years, 65% in age group 45–65 years and 42% of more than 65 years belonged to the extended symptomatic subgroup in the study cohort. Though the number of cases more than 65 years age was less (12 symptomatic), overall the responses were near similar to young age groups. Also, we observed a somewhat lower response for IFN-γ. The CHIKV IgG antibody response in elderly appeared robust.

## Discussion

This report describes the serological cytokine phenotype of acute CHIKV based on a rural community survey carried out during the major CHIKV epidemic in India in 2006. An intensely up regulated Th1 (IFN –α, -β and -γ, TNF-α, IL −1β, CXCL-10/IP-10, and MCP-1) and Th2 (IL-4, IL-6, IL-10 and IL-13) cytokines was demonstrated both in the symptomatic and recovered subjects within four weeks of onset of illness. While the Th1 cytokines were found maximal during the acute phase (<6 days), Th2 cytokines appeared to peak in subjects with extended symptomatic phase (15–30 days). Though several cytokines remained persistently elevated in the asymptomatic subjects during the study period, the level was lower than that observed in symptomatic subjects. The cytokine repertoire phenotype in serologically confirmed cases of CHIKV (Table S10 in File S1, Figure S1) was almost similar to that observed in study cohort (Table 2, Figure 2). Several significant (p<0.05) and robust (moderate to strong) correlates of cytokines pertaining to clinical (pain, myalgias, fatigue, headaches), laboratory (blood hemoglobin, leukocyte and platelet counts, CRP ) and antibody (anti CHIKV IgM and IgG) parameters were described. Increased ESR at initial evaluation and persistently elevated CRP (Table 1) indicated an intense systemic inflammatory response by the CHIKV virus that spilled over beyond the acute illness phase.

The detail clinical and immunological profile of CHIKV in our rural survey has been reported earlier [1], [2]. However, some observations may be relevant to the current cytokine phenotype. It is prudent to state that this study was designed a-priori to the rural population survey. The survey was prompted by several clinically important observations of post CHIKV arthritis and rheumatism made by us from a popular rheumatology outpatient clinic [8]. The survey team headed by a rheumatologist (AC) and microbiologist (AV) worked in field conditions to complete the survey and collect blood samples. Thus a large spectrum of data of patients with varying duration of illness beginning with the very acute phase was captured. Overall, unlike several published studies with retrospective design, the current cross-sectional and prospective design study pertain to real life scenario in acute CHIKV.

It is uncommon to detect IgM within 4 days of illness [13] and unusual to demonstrate IgG in acute phase [14] **.** In the current acute illness subjects, 17% showed anti IgM and 52% showed IgG antibodies to CHIKV. We have earlier validated the current study technique of IgM detection by comparing it with standard Mac ELISA [1]. 51% and 40% patients in the current cohort were seropositive for anti CHIKV IgM and IgG antibodies. The phase of viraemia is generally over within a week of onset of illness [15], [5]. During the Indian epidemic (2006), 37.3% cases were reported positive for anti-CHIKV IgM antibody [13]. 35.7%, 4.9% and 0.4% of the sera samples respectively collected from 473 cases, predominantly rural, tested positive for anti-CHIKV IgM, anti-dengue IgM, and anti-CHIKV plus anti-dengue IgM response respectively in another Indian study during the epidemic [3]. 48.6%, 55.4%, 21.5% and 62.5% blood samples respectively tested positive for CHIKV using RT-PCR (reverse transcriptase-polymerase chain reaction), RT-LAMP (reverse transcriptase light isothermal loop amplification), IgM (ELISA) and virus isolation technique respectively in 296 clinically suspected patients referred to a tertiary medical center; time of collection of blood sample with respect to onset of illness was not described [16]. We could not find published data on serum anti CHIKV IgG antibodies during the early phase of illness. The very early switch from only IgM to IgM and IgG response to CHIKV shown in the current study is intriguing and not described to the best of our knowledge. It is likely that this early antibody response was preceded by an intense early Th2 cytokine up regulation (Table 2). However, a large number of patients with typical acute illness and intense Th1 and Th2 cytokine response did not test seropositive for anti CHIKV antibodies and needs to be investigated further. Our regression model to predict early clinical remission of the musculoskeltal symptoms did not identify anti CHIKV IgM response as a predictor of extended symptomatic phase (delay resolution). 50% recovered cases were seropositive for anti CHIKV IgG antibody (Table 1). Only 13% extended symptomatic patients tested seropositive for anti CHIKV IgG and this suggests the lack of specific IgG response with ‘prolonged/extended symptoms (MSK)’ in CHIKV. Overall, the cytokine rise in the current study (except IL-4 and IL-10) was much more in subjects seronegative for specific anti CHIKV antibodies (Tables in File S1) and suggests an immune host response to control the illness. A recent study, using mouse model, demonstrated an essential role for B cells and specific antibodies in controlling CHIKV infection [17].

We demonstrated an early intense interferon response (IFN-α, -β, -γ) which persisted in patients with extended symptoms. Persistent interferon response is likely to be associated with a failure to eradicate virus by the host at least during the current study period of four week post illness onset. As shown in Table 3, significant positive correlation was observed between IFN-β and fatigue, IFN-γ and low backache and IFN-γ and pain intensity; IFN-α and –β both showed inverse correlation with myalgia. Both Th1 and Th2 cytokines were intensely elevated (Table 2, Fig. 2) during all illness phases and recovered group with varying intensity. Interestingly, both Th1 and Th2 cytokines demonstrated a highly intense or maximum response in the subgroup with persistent musculoskeletal symptoms beyond 14 days of illness; only exception being a conspicuous drop in IL-4 assay. Another added feature was a relatively low IgG (anti CHIKV) antibody response (Table 1). All this perhaps indicates a somewhat failed host response to curb the virus in a landscape marked by heightened activation of T cells and macrophages and lesser intense B cell activation. The strong negative correlations (Table 4) of the Th2 cytokines (IL 4 and IL 6) with those of interferon response (Th1 proinflammatory cytokines) probably show an attempt by the cellular, humoral and immune events to curtail the Th1 cytokine reactivity and stop the progression of virus in the host.

Rapid induction of type I interferon (IFN) expression is critical to stimulating a robust innate immune response against viral infection and requires the activation of multiple transcriptional proteins following engagement and signaling through Toll-like receptor-dependent and independent pathways [18]. Alpha viruses, like several other viruses are potent inducers of interferon which in turn inhibits viral replication. Gifford and Heller [19] reported for the first time during the 1954–1964 CHIKV epidemic in Asia and South India, that chick embryo fibroblasts infected with CHIKV produced detectable levels of type 1 interferon three hours after infection. Macrophages, a major source of interferon, are readily infected by CHIKV and play a pivotal role in several cellular responses shown in experimental studies [20], [21] and in both Th1 and Th2 cytokine response. Based on the observation of a sharp decline in viraemia before the appearance of high-affinity neutralizing antibodies, it was hypothesized that type 1 IFNs mediate antiviral response [22]. Type II IFN (IFN-γ) produced in early CHIKV infection further promotes the transition from innate to adaptive immunity [22]. An earlier study had shown that CHIKV-specific CD4+ T cells induced by CHIKV infection were the major producers of IFN- γ and that Th1 cells were probably responsible for a skewed production of IgG2 antibodies by B cells in response to IFN- γ [23]. IFN-gamma is made exclusively by natural killer and T cells and has important immunoregulatory functions including intense stimulation of macrophages, monocytes, fibroblasts and dendritic cells.

Pro-inflammatory mediators including TNF-α, MCP-1, IFN- γ, IFN- α/β and IL-6 have been identified in primates and humans infected with CHIKV and in other alphaviral arthritides [24], [25], [26]. Immunological studies on muscle biopsies from CHIKV infected patients with myositis syndrome showed presence of viral antigens located exclusively inside skeletal muscle progenitor cells and not in muscle fibres [27]. Though pathophysiologically, the intense and conjoint upregulation of Th1 and Th2 cytokines definitely contribute to the severe but self limiting profile of painful inflammatory pattern of the musculoskeletal illness in CHIKV, the literature is silent on the individual contribution of cytokines towards the clinical phenotype. In this report (Table 2 and tables S1–S8 in File S1) we have attempted to describe interesting and plausible clinical, laboratory and serological (antibody) correlations of the up-regulated cytokines.

To the best of our knowledge, this is the first report to describe IL-13 responses in acute CHIKV. While IFN – γ, TNF- α and CXCL-10/IP-10 are pro-inflammatory and a hallmark of Th1 response, IL-13 (strikingly homologous to IL-4) is a signature cytokine of the Th2 response and several subjects in the current study belonging to different symptomatic subgroups and recovered group showed intense elevation (Fig. 2). IL-13 is considered to an important immunomodulatory cytokine which also induces B cell proliferation and IgE switching. IL-13 was reported to play an important role in the hypersensitive and allergy responses in the respiratory tract and gut (nematode infestations) and is implicated with fibrosis in scleroderma [28]. In the current study, there were no differences in the IL-13 assay results obtained in the acute, sub acute and convalescent cases. We are not able to find a satisfactory explanation for persistent heightened IL-13 response in the study. We speculate that IL-13 may be associated with persistent arthritis in CHIKV.

Several other researchers have published data on cytokines in CHIKV [29], [30], [31], [32]. We have chosen some of the studies for comparison (Table 5). It is prudent to add that precise comparison is not appropriate for several reasons pertaining to small sample size, timing of blood collection and assay methods. Our focus was on cytokines in acute illness and persistence of musculoskeletal symptoms from 14–30 days after onset of illness (extended symptomatic group). Increased IL-1, IL-10 and IFN-γ assay reported suggests an early engagement of adaptive immunity [33]. On comparison, discrepant results were observed in the study reported by Kelvin et al (2011) where the median levels of IL-4 and IL-10 was lower in the acute cases than in the healthy controls. Increased levels (1.5 times higher than controls) of IL-2R was reported in acute CHIKV cases [25], [31]. Also, Chaaithanya et al (2011) have also reported high levels of IL-1 receptor antagonist in acute CHIKV cases. Increased levels of several other cytokines (IFN-α, CXCL-9, CCL2, IL-8 and IL-17 have been reported [25], [30], [31].

**Table 5:** Comparison of the cytokine profile in CHIKV cases with healthy controls in different studies reported.

Cytokine/Chemokine	Current Study (HC = 80)			Ng et al 2009 (HC = 9)	Kelvin et al 2011 (HC = 10)	Chaaithanya et al 2011 (HC = 6)	El-Gabalawy et al 2012 (HC = 200)
	Acute(n = 23)	Subacute(n = 22)	Extended Symptomatic(n = 65)	2–19 days(n = 10)	Acute(n = 35)	Acute(n = 6)	RA(n = 105)
IFN-α	8.9	3.6	3.0	1.5	ND	ND	2.8
IFN-β	8.4	3.5	2.9	ND	ND	ND	ND
IFN-γ	50.1	17.2	15.8	+	0.9	ND	2.7
CXCL-10/IP-10	26.9	23.3	25.0	1000	2.3	33.9	1.2
IL1-β	2.3	1.7	1.7	+	0.82	ND	4.9
TNF-α	4.4	4.6	5.2	+	1.5	ND	3.7
MCP-1	49.0	37.9	33.2	ND	ND	1.9	1.1
IL-4	108.7	392.6	170.5	+	0.07	ND	6.5
IL-6	3.8	7.0	7.5	4.0	2.0	196.7	9.3
IL-10	6.4	23.1	38.5	1.5	0.4	1.9	3.3
IL-13	3.3	2.5	3.2	+	ND	ND	10

*Values expressed as ratio of medians of cases to controls.*

IFN-Interferon; TNF = Tumor Necrosis Factor; CXCL-10/IP-10 = Interferon Gamma-Induced Protein-10; IL = Interleukin; MCP = Monocyte Chemoattractant Protein. HC = Healthy controls; ND = Not Done;+ = Higher than Healthy controls; **Note**: In the current study, IFN-α, IFN-β, IL-1β, MCP-1, IL-4 and IL-10 were tested in 4 subjects of sub-acute phase and of extended symptomatic phase. All other cytokines were tested in 23, 22 and 65 subjects of acute, sub-acute and extended symptomatic phase respectively.

In Table 5, we have compared studies by computing a ratio of median value of cytokine obtained in illness to that in healthy subjects (control). We used published data and we chose acute or early illness. The ratio of several cytokines in the current study was several fold higher than that calculated for other studies. To provide an interesting perspective to the intense cytokine phenotype in the current study (Table 5) we also included a similar cytokine assay ratio profile from a study of patients suffering from Rheumatoid Arthritis (RA) [34] which is a prototype of an intense immune inflammatory disorder. However, unlike CHIKV, RA is a chronic disorder and an upregulation of immune cytokine response is reported several years prior to onset of the clinical disease [35]. Infrequently, CHIKV can lead to chronic persistent painful MSK illness and arthritis and was the subject of our earlier publications [1], [8].

The current study had several limitations. Compared to the large number of clinically identified cases, much lesser number of cases consented to donate blood samples. Cases identified during the survey had varying period of illness and in several the clinical diagnosis was based on historical narration. We did not use concurrent controls but chose blood samples collected a year earlier to CHIKV epidemic to avoid sub-clinical illness during the epidemic (see methods section). Despite the fact, that we made frequent visits to the study village during the epidemic period, we managed to collect blood samples of only 23 acute cases. Anti CHIKV IgG assay was only carried out in limited number of cases. We faced several difficult logistic issues including prolonged and cumbersome travel between our center (CRD) and the village. We were unable to carry out tests for viral RNA detection and viral load. However, the relevance of the study was increased by the fact that a well defined rural population was meticulously screened during the epidemic as per a strict protocol. A large number of clinical cases were evaluated by a an investigation team (CRD) that has been engaged in epidemiological studies in the region since 1996.

We have followed our survey cases for periods ranging from 12–24 months and repeatedly collected blood samples for cytokines assay and other relevant autoantibodies (rheumatic diseases) in a prospective study. Our principal focus was on persistent chronic arthritis and a separate report will describe the results. Meanwhile, we have published two studies [1], [36] to demonstrate persistence of IL-6 upto 18 months of follow-up of cases with chronic musculoskeletal symptoms and minimal or nil effect of chloroquin on selected Th1 and Th 2 cytokines in a 24 week randomized blind control drug trial study.

This is the first comprehensive clinical report on the pathophysiology of acute CHIKV based on Th1 and Th2 cytokines and anti CHIKV antibodies based on a rural population survey during the 2006 Indian epidemic. An early and intense up regulation of number of pro-inflammatory and anti inflammatory cytokines was demonstrated during the early period in subjects with a typical self limiting illness. However, this phenomenon, albeit a lesser intensity, was also evident in cases with extended symptoms and in asymptomatic individuals. An early specific IgM and IgG antibody response to CHIKV was also seen in a significant number of cases. Early persistent IgM and lower IgG to anti CHKV and intense Th2 cytokine phenotype seem to be associated with delay in resolution and prolonged MSK symptoms. Interestingly, maximum assay of TNF-α, IL-6 and IL-13 with low anti CHIKV IgM response were found in subjects recovered from CHIKV within one month of illness onset. Though we are able to provide reasonable explanation on the likely associations of several upregulated cytokines (Th1 and Th2) with the clinical phenotype and specific anti CHIKV antibody response (IgM and IgG), a more comprehensive work-up of virus markers, cytokines and immune responses (cellular and humoral) would be required to establish concrete causal and contributory relationships.

## Supporting Information

Figure S1 **Scatter graphs of cytokines of anti CHIKV seropositive (IgM and/or IgG) versus seronegative (IgM and IgG) in symptomatic cases. Footnote to Supplementary** **Figure 1:** IFN-Interferon; TNF = Tumor Necrosis Factor; CXCL-10/IP-10 = Interferon Gamma-Induced Protein 10; IL = Interleukin; MCP = Monocyte Chemoattractant Protein. Statistical analysis was performed using non-parametric Kruskal Wallis test. Significant differences between the two groups was observed in IFN-α, IFN-β, IL-1β, IL-4 and IL-10.
(TIF)

File S1 **Supporting tables. Table S1**, Comparison of cytokine profile of CHIKV IgM negative versus IgM positive in acute Cases (N = 23). **Table S2**, Comparison of cytokine profile of CHIKV IgG negative versus IgG positive in acute cases (N = 23). **Table S3**, Comparison of cytokine profile of CHIKV IgM &/or IgG negative versus IgM &/or IgG positive in acute cases (N = 23). **Table S4**, Comparison of cytokine profile of CHIKV IgM negative versus IgM positive in subacute cases. **Table S5**, Comparison of cytokine profile of CHIKV IgM negative versus IgM positive in extended subacute cases. **Table S6**, Comparison of cytokine profile of CHIKV IgM negative versus IgM positive in recovered cases (N = 22). **Table S7**, Comparison of cytokine profile of CHIKV IgM negative versus IgM positive in symptomatic cases. **Table S8**, Comparison of cytokine profile of CHIKV IgM positive versus IgG positive in symptomatic cases. **Table S9**, Comparison of serological profile (frequency of positive result) and cytokine profile (medians) of symptomatic cases (N = 110) and recovered cases (N = 22) of CHIKV with illness within one month duration between age groups. **Table S10**, Comparison of cytokine profile of anti CHIKV seropositive (IgM and/or IgG) versus seronegative (IgM and IgG) in symptomatic cases.
(DOCX)
