# Supplementary figures and images for: Cytokines in Acute Chikungunya
Source: PLoS One. 2014 Oct 24;9(10):e111305. doi: 10.1371/journal.pone.0111305 (PMC4208842; doi:10.1371/journal.pone.0111305)

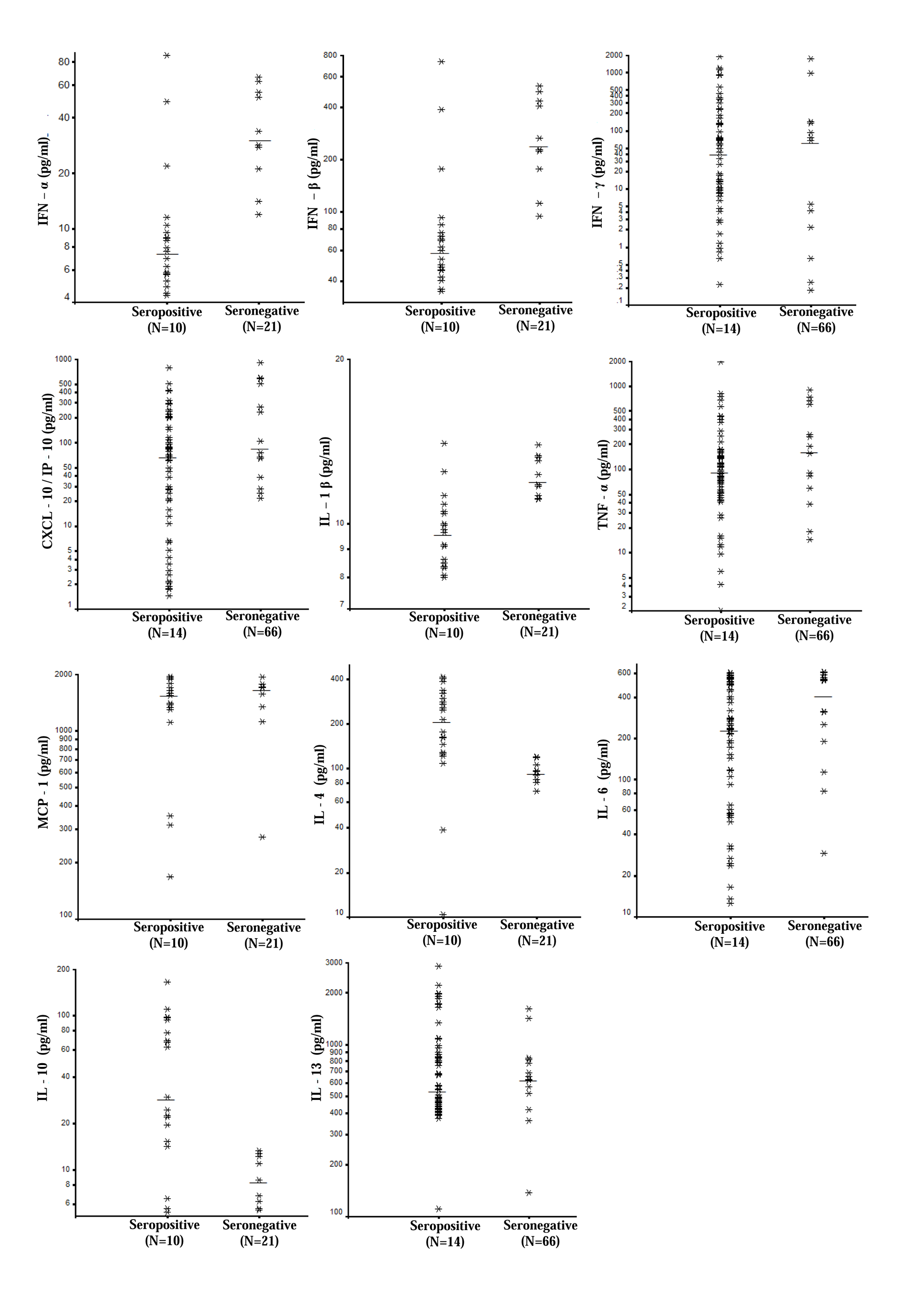

Supplement: Figure S1 — Scatter graphs of cytokines of anti CHIKV seropositive (IgM and/or IgG) versus seronegative (IgM and IgG) in symptomatic cases. Footnote to Supplementary Figure 1: IFN-Interferon; TNF = Tumor Necrosis Factor; CXCL-10/IP-10 = Interferon Gamma-Induced Protein 10; IL = Interleukin; MCP = Monocyte Chemoattractant Protein. Statistical analysis was performed using non-parametric Kruskal Wallis test. Significant differences between the two groups was observed in IFN-α, IFN-β, IL-1β, IL-4 and IL-10. (TIF) [file pone.0111305.s001.tif]
