# Supplementary material for: Cytokines in Acute Chikungunya
Source: PLoS One. 2014 Oct 24;9(10):e111305. doi: 10.1371/journal.pone.0111305 (PMC4208842; doi:10.1371/journal.pone.0111305)
Supplement: File S1 — Supporting tables. Table S1, Comparison of cytokine profile of CHIKV IgM negative versus IgM positive in acute Cases (N = 23). Table S2, Comparison of cytokine profile of CHIKV IgG negative versus IgG positive in acute cases (N = 23). Table S3, Comparison of cytokine profile of CHIKV IgM &/or IgG negative versus IgM &/or IgG positive in acute cases (N = 23). Table S4, Comparison of cytokine profile of CHIKV IgM negative versus IgM positive in subacute cases. Table S5, Comparison of cytokine profile of CHIKV IgM negative versus IgM positive in extended subacute cases. Table S6, Comparison of cytokine profile of CHIKV IgM negative versus IgM positive in recovered cases (N = 22). Table S7, Comparison of cytokine profile of CHIKV IgM negative versus IgM positive in symptomatic cases. Table S8, Comparison of cytokine profile of CHIKV IgM positive versus IgG positive in symptomatic cases. Table S9, Comparison of serological profile (frequency of positive result) and cytokine profile (medians) of symptomatic cases (N = 110) and recovered cases (N = 22) of CHIKV with illness within one month duration between age groups. Table S10, Comparison of cytokine profile of anti CHIKV seropositive (IgM and/or IgG) versus seronegative (IgM and IgG) in symptomatic cases. (DOCX) [file pone.0111305.s002.docx]

**Table S1: Comparison of cytokine profile of CHIKV IgM negative versus IgM positive in acute Cases (N=23).** *Values expressed as median [standard deviation](interquartile range) pg/ml.*

| **Cytokine**  **(pg/ml)** | Anti-CHIKV IgM Negative  N=19 | Anti-CHIKV IgM Positive  N=4 | ‘p’ |
| --- | --- | --- | --- |
| IFN-α | 21.88  [24.10]  (10.50 – 51.52) | 8.29  [0.92]  (7.18 – 8.93) | 0.02 |
| IFN-β | 177.23  [197.02]  (84.21 – 407.72) | 65.88  [8.26]  (56.04 – 71.75) | 0.02 |
| IFN-γ | 77.36  [511.79]  (42.88 – 65.13) | 242.8  [212.59]  (8.71 – 433.13) | 0.79 |
| CXCL-10 / IP-10 | 100.36  [291.86]  (25.08 – 504.40) | 5.91  [69.85]  (2.60 – 109.78) | 0.04 |
| IL-1β | 11.27  [1.42]  (10.56 – 13.02) | 9.86  [0.39]  (9.33 – 10.00) | 0.01 |
| TNF-α | 140.03  [458.98]  (38.20 – 251.68) | 24.18  [22.48]  (3.03 – 43.04) | 0.02 |
| MCP-1 | 1605.09  [510.60]  (1333.64 – 1810.12) | 1629.50  [753.73]  (79.88 – 1927.49) | 0.83 |
| IL-4 | 107.40  [74.28]  (83.60 – 126.40) | 271.93  [77.71]  (171.64 – 316.3) | 0.01 |
| IL-6 | 191.58  [230.74]  (32.92 – 537.52) | 38.67  [230.51]  (18.52 – 381.65) | 0.12 |
| IL-10 | 11.65  [42.53]  (6.08 – 14.44) | 59.12  [44.05]  (10.14 – 97.33) | 0.03 |
| IL-13 | 519.24  [547.34]  (403.43 – 780.05) | 671.98  [88.54]  (603.20 – 766.37) | 0.33 |

IFN-Interferon; TNF=Tumor Necrosis Factor; CXCL-10/IP-10=Interferon Gamma-Induced protein-10; IL=interleukin; MCP=Monocyte Chemoattractant Protein. Statistical analysis was performed using non-parametric Kruskal Wallis test.

**Table S2: Comparison of cytokine profile of CHIKV IgG negative versus IgG positive in acute cases (N=23).** *Values expressed as median [standard deviation](interquartile range) pg/ml..*

| **Cytokine**  **(pg/ml)** | Anti-CHIKV IgG Negative  N=11 | Anti-CHIKV IgG Positive  N=12 | ‘p’ |
| --- | --- | --- | --- |
| IFN-α | 28.42  [20.94]  (14.16 – 54.80) | 9.31  [24.34]  (7.86 – 19.31) | 0.12 |
| IFN-β | 227.99  [166.32]  (112.57 – 437.88) | 74.40  [203.79]  (62.33 – 156.20) | 0.02 |
| IFN-γ | 113.82  [560.27]  (52.60 – 571.52) | 72.71  [402.68]  (15.93 – 495.21) | 0.65 |
| CXCL-10 / IP-10 | 105.51  [315.15]  (24.88 – 585.63) | 50.57  [234.10 ]  (8.26 – 190.22) | 0.27 |
| IL-1β | 11.80  [1.24]  (11.15 – 13.25) | 10.23  [1.41]  (9.73 – 11.16) | 0.02 |
| TNF-α | 90.99  [259.86]  (18.01 – 259.99) | 97.83  [548.54]  (20.00 – 145.63) | 0.58 |
| MCP-1 | 1694.49  [500.29]  (1287.90 – 1813.84) | 1558.54  [593.03]  (1318.55 – 19.5.14) | 0.67 |
| IL-4 | 96.36  [73.33]  (83.60 – 119.26) | 137.94  [97.78]  (111.88 – 267.36) | 0.07 |
| IL-6 | 310.52  [224.96]  (82.64 – 542.71) | 45.14  [234.54]  (25.05 – 494.88) | 0.09 |
| IL-10 | 9.82  [5.07]  (6.08 – 12.98) | 17.44  [54.46]  (8.44 – 97.33) | 0.04 |
| IL-13 | 629.37  [439.05]  (420.15 – 780.05) | 545.18  [563.52]  (408.26 – 787.75) | 0.85 |

IFN-Interferon; TNF=Tumor Necrosis Factor; CXCL-10/IP-10=Interferon Gamma-Induced protein-10; IL=interleukin; MCP=Monocyte Chemoattractant Protein. Statistical analysis was performed using non-parametric Kruskal Wallis test.

**Table S3: Comparison of cytokine profile of CHIKV IgM &/or IgG negative versus IgM &/or IgG positive in acute cases (N=23).** *Values expressed as median [standard deviation](interquartile range) pg/ml.*

| **Cytokine**  **(pg/ml)** | Anti-CHIKV IgM &/or IgG Negative  N=11 | Anti-CHIKV IgM &/or IgG Positive  N=13 | ‘p’ |
| --- | --- | --- | --- |
| IFN-α | 31.02  [20.00]  (19.43 – 56.77) | 9.02  [23.53]  (7.76 – 16.74) | 0.004 |
| IFN-β | 247.48  [158.69]  (161.07 – 451.82) | 72.79  [196.99]  (61.67 – 135.18) | 0.004 |
| IFN-γ | 93.11  [593.73]  (36.91 – 566.02) | 74.06  [384.53]  (18.34 – 433.13) | 0.97 |
| CXCL-10 / IP-10 | 187.49  [317.96]  (27.07 – 588.57) | 29.70  [227.72]  (5.91 – 174.87) | 0.09 |
| IL-1β | 12.06  [1.03]  (11.24 – 13.27) | 10.02  [1.37]  (9.82 – 11.06) | 0.004 |
| TNF-α | 123.27  [264.83]  (33.15 – 363.02) | 80.92  [529.42]  (12.20 -144.57) | 0.24 |
| MCP-1 | 1694.00  [505.06]  (1231.06 – 1741.50) | 1576.41  [586.27]  (1336.76 – 1916.55) | 0.82 |
| IL-4 | 95.42  [16.85]  (82.61 – 112.53) | 146.53  [102.77]  (115.10 – 276.55) | 0.01 |
| IL-6 | 313.11  [220.27]  (106.17 – 545.45) | 52.82  [228.34]  (25.58 – 493.67) | 0.05 |
| IL-10 | 8.61  [3.26]  (5.93 – 12.56) | 19.56  [52.63]  (10.35 – 97.01) | 0.02 |
| IL-13 | 598.89  [462.64]  (405.34 – 942.01) | 581.34  [539.83]  (413.09 – 778.74) | 1.00 |

IFN-Interferon; TNF=Tumor Necrosis Factor; CXCL-10/IP-10=Interferon Gamma-Induced protein-10; IL=interleukin; MCP=Monocyte Chemoattractant Protein. Statistical analysis was performed using non-parametric Kruskal Wallis test.

**Table S4: Comparison of cytokine profile of CHIKV IgM negative versus IgM positive in subacute cases.** *Values expressed as median [standard deviation](interquartile range) pg/ml.*

| **Cytokine**  **(pg/ml)** | Anti-CHIKV IgM Negative | | Anti-CHIKV IgM Positive | | ‘p’ |
| --- | --- | --- | --- | --- | --- |
|  | N | Value | N | Value |  |
| IFN-α | 1 | 5.26 | 3 | 5.74  [0.08]  (5.68 – 5.84) | 0.18 |
| IFN-β | 1 | 42.16 | 3 | 46.50  [1.19]  (45.99 – 48.26) | 0.18 |
| IFN-γ | 10 | 38.44  [60.89]  (12.67 – 85.27) | 12 | 12.38  [276.22]  (4.63 – 57.40) | 0.29 |
| CXCL-10 / IP-10 | 10 | 56.09  [152.95]  (4.09 – 104.22) | 12 | 61.81  [87.15]  (22.83 – 93.84) | 1.00 |
| IL-1β | 1 | 8.37 | 3 | 8.62  [0.32]  (8.50 – 9.11) | 0.18 |
| TNF-α | 10 | 157.03  [760.29]  (81.73 – 712.17) | 12 | 87.92  [144.79]  (53.72 – 118.13) | 0.10 |
| MCP-1 | 1 | 1700.84 | 3 | 1107.21  [532.54]  (317.01 – 1330.55) | 0.18 |
| IL-4 | 1 | 401.51 | 3 | 383.62  [39.01]  (335.28 – 412.56) | 0.66 |
| IL-6 | 10 | 232.03  [150.33]  (90.19 – 315.83) | 12 | 233.74  [140.25]  (198.01 – 279.56) | 0.60 |
| IL-10 | 1 | 62.44 | 3 | 29.78  [23.56]  (24.52 – 67.71) | 0.66 |
| IL-13 | 10 | 501.35  [464.22]  (440.44 – 832.52) | 12 | 435.48  [199.66]  (412.31 – 747.91) | 0.17 |

IFN-Interferon; TNF=Tumor Necrosis Factor; CXCL-10/IP-10=Interferon Gamma-Induced protein-10; IL=interleukin; MCP=Monocyte Chemoattractant Protein. Statistical analysis was performed using non-parametric Kruskal Wallis test.

**Table S5: Comparison of cytokine profile of CHIKV IgM negative versus IgM positive in extended subacute cases.** *Values expressed as median [standard deviation](interquartile range) pg/ml.*

| **Cytokine**  **(pg/ml)** | Anti-CHIKV IgM Negative | | Anti-CHIKV IgM Positive | | ‘p’ |
| --- | --- | --- | --- | --- | --- |
|  | N | Value | N | Value |  |
| IFN-α | ND | - | 4 | 4.68  [0.40]  (4.40 – 5.16) | - |
| IFN-β | ND | - | 4 | 38.11  [3.43]  (35.28 – 41.70) | - |
| IFN-γ | 25 | 26.84  [164.14]  (5.89 - 89.00) | 40 | 30.11  [399.47]  (9.23 – 148.61) | 0.61 |
| CXCL-10 / IP-10 | 25 | 17.69  [68.34]  (4.28 – 87.08) | 40 | 84.04  [104.05]  (12.03 – 114.13) | 0.10 |
| IL-1β | ND | - | 4 | 8.19  [0.18]  (8.02 – 8.36) | - |
| TNF-α | 25 | 96.41  [272.61]  (50.65 – 436.77) | 40 | 108.56  [200.85]  (57.39 – 174.98) | 0.71 |
| MCP-1 | ND | - | 4 | 1869.23  [778.26]  (711.79 – 1955.56) | - |
| IL-4 | ND | - | 4 | 170.53  [24.26]  (162.01 – 204.77) | - |
| IL-6 | 25 | 184.01  [210.58]  (55.74 – 487.85) | 40 | 262.00  [190.73]  (116.70 – 500.60) | 0.37 |
| IL-10 | ND | - | 4 | 73.00  [11.87]  (68.90 – 89.82) | - |
| IL-13 | 25 | 613.44  [1315.15]  (461.33 – 1573.42) | 40 | 575.38  [601.49]  (460.75 – 1090.00) | 0.91 |

IFN-Interferon; TNF=Tumor Necrosis Factor; CXCL-10/IP-10=Interferon Gamma-Induced protein-10; IL=interleukin; MCP=Monocyte Chemoattractant Protein; ND=Not Done. Statistical analysis was performed using non-parametric Kruskal Wallis test.

**Table S6: Comparison of cytokine profile of CHIKV IgM negative versus IgM positive in recovered cases (N=22).** *Values expressed as median [standard deviation](interquartile range) pg/ml.*

| **Cytokine**  **(pg/ml)** | Anti-CHIKV IgM Negative  N=15 | Anti-CHIKV IgM Positive  N=7 | ‘p’ |
| --- | --- | --- | --- |
| IFN-γ | 38.11  [71.04]  (0.62 – 123.63) | 5.82  [120.93]  (0.94 – 119.40) | 0.73 |
| CXCL-10 / IP-10 | 67.95  [132.34]  (29.70 – 165.66) | 115.64  [53.06]  (43.24 – 147.97) | 0.60 |
| TNF-α | 245.90  [508.84]  (59.01 – 607.02) | 269.86  [433.45]  (102.62 – 912.01) | 0.75 |
| IL-6 | 454.90  [220.44]  (92.07 – 545.79) | 546.15  [138.19]  (444.76 – 578.58) | 0.22 |
| IL-13 | 760.74  [1581.29]  (585.42 – 844.63) | 749.03  [126.66]  (698.84 – 785.11) | 0.92 |

IFN-Interferon; TNF=Tumor Necrosis Factor; CXCL-10/IP-10=Interferon Gamma-Induced protein-10; IL=interleukin; MCP=Monocyte Chemoattractant Protein; Statistical analysis was performed using non-parametric Kruskal Wallis test.

.

**Table S7: Comparison of cytokine profile of CHIKV IgM negative versus IgM positive in symptomatic cases. .** *Values expressed as median [standard deviation](interquartile range) pg/ml.*

| **Cytokine**  **(pg/ml)** | Anti-CHIKV IgM Negative | | Anti-CHIKV IgM Positive | | ‘p’ |
| --- | --- | --- | --- | --- | --- |
|  | N | Value | N | Value |  |
| IFN-α | 20 | 21.52  [24.14]  (9.83 – 50.89) | 11 | 5.74  [1.64]  (4.87 – 7.93) | 0.00 |
| IFN-β | 20 | 177.23  [197.21]  (78.05 – 403.39) | 11 | 46.50  [12.88]  (40.34 – 63.11) | 0.00 |
| IFN-γ | 54 | 54.27  [333.53]  (12.38 – 118.56) | 56 | 22.45  [365.80]  (8.90 – 157.21) | 0.48 |
| CXCL-10 / IP-10 | 54 | 47.60  [207.26]  (6.56 – 110.83) | 56 | 70.25  [98.66]  (7.69 – 106.74) | 0.97 |
| IL-1β | 20 | 11.27  [1.57]  (10.47 – 12.88) | 11 | 8.62  [0.75]  (8.32 – 9.78) | 0.00 |
| TNF-α | 54 | 141.24  [460.30]  (59.07 – 292.56) | 56 | 94.15  [186.19]  (51.98 – 147.45) | 0.13 |
| MCP-1 | 20 | 1633.77  [498.97]  (1344.74 – 1776.29) | 11 | 1373.19  [687.13]  (354.01 – 1941.39) | 0.97 |
| IL-4 | 20 | 108.67  [96.46]  (84.59 – 129.34) | 11 | 246.95  [95.10]  (164.04 – 335.28) | 0.001 |
| IL-6 | 54 | 208.76  [205.72]  (57.01 – 471.47) | 56 | 243.90  [184.36]  (108.21 – 434.35) | 0.47 |
| IL-10 | 20 | 12.28  [42.24]  (6.23 – 15.31) | 11 | 68.90  [31.09]  (24.52 – 94.08) | 0.001 |
| IL-13 | 54 | 556.68  [980.86]  (440.44 – 835.81) | 56 | 565.00  [534.86]  (439.83 – 900.41) | 0.80 |

IFN-Interferon; TNF=Tumor Necrosis Factor; CXCL-10/IP-10=Interferon Gamma-Induced Protein-10; IL=Interleukin; MCP=Monocyte Chemoattractant Protein.

Statistical analysis was performed using non-parametric Kruskal Wallis test.

**Table S8:** **Comparison of cytokine profile of CHIKV IgM positive versus IgG positive in symptomatic cases. .** *Values expressed as median [standard deviation](interquartile range) pg/ml.*

| **Cytokine**  **(pg/ml)** | Anti-CHIKV IgM Positive | | Anti-CHIKV IgG Positive | | ‘p’ |
| --- | --- | --- | --- | --- | --- |
|  | N | Value | N | Value |  |
| IFN-α | 5 | 4.87  [1.46]  (4.43 – 6.59) | 10 | 10.05  [26.42]  (7.26 – 28.66) | 0.01 |
| IFN-β | 5 | 40.34  [11.46]  ()35.48 – 52.63 | 10 | 80.10  [221.20]  (57.60 – 230.53) | 0.01 |
| IFN-γ | 11 | 157.08  [599.96]  (5.13 – 555.48) | 10 | 74.06  [420.38]  (42.88 – 625.13) | 0.94 |
| CXCL-10 / IP-10 | 11 | 204.18  [141.01]  (38.47 – 297.59) | 10 | 85.90  [267.98]  (22.08 – 439.55) | 0.83 |
| IL-1β | 5 | 8.32  [0.73]  (8.04 – 9.08) | 10 | 10.50  [1.63]  (9.52 – 11.56) | 0.01 |
| TNF-α | 11 | 97.20  [225.57]  (57.39 – 212.19) | 10 | 141.24  [590.94]  (63.82 – 181.35) | 0.94 |
| MCP-1 | 5 | 1941.39  [699.12]  (1069.57 – 1954.82) | 10 | 1605.09  [519.28]  (1381.37 – 1914.07) | 0.13 |
| IL-4 | 5 | 177.03  [67.44]  (162.68 – 268.21) | 10 | 127.38  [122.92]  (91.25 – 273.78) | 0.33 |
| IL-6 | 11 | 495.22  [168.87]  (273.83 – 550.50) | 10 | 114.88  [237.75]  (26.80 – 500.41) | 0.07 |
| IL-10 | 5 | 68.90  [26.80]  (45.39 – 85.56) | 10 | 14.74  [54.64]  (6.31 – 74.25) | 0.11 |
| IL-13 | 11 | 811.08  [779.69]  (671.79 – 1653.46) | 10 | 503.15  [620.26]  (395.99 – 1097.11) | 0.12 |

IFN-Interferon; TNF=Tumor Necrosis Factor; CXCL-10/IP-10=Interferon Gamma-Induced protein-10; IL=Interleukin; MCP=Monocyte Chemoattractant Protein. Statistical analysis was performed using non-parametric Kruskal Wallis test.

.

**Table S9: Comparison of serological profile (frequency of positive result) and cytokine profile (medians) of symptomatic cases (N=110) and recovered cases (N=22) of CHIKV with illness within one month duration between age groups.**

| **Test** | **Less than 20 years** | | **21 – 44 years** | | **45 – 65 years** | | **More than 65** | |
| --- | --- | --- | --- | --- | --- | --- | --- | --- |
|  | **Symptomatic**  **N=16** | **Recovered**  **N=2** | **Symptomatic**  **N=45** | **Recovered**  **N=12** | **Symptomatic**  **N=37** | **Recovered**  **N=6** | **Symptomatic**  **N=12** | **Recovered**  **N=2** |
| CHIKV IgM+ | 8 (50%) | 0 | 26 ((58%) | 5 (42%) | 17 (46%) | 1 (17%) | 5 (42%) | 1 (50%) |
| CHIKV IgG+ | 1/3 (33%) | 0/1 | 10/19 (53%) | 6/9 (67%) | 3/16 (19%) | 1/4 (25%) | 3/4 (75%) | 1/2 (50%) |
| IFN-α | 27.73 | ND | 8.90 | ND | 28.42 | ND | 8.72 | ND |
| IFN-β | 223.94 | ND | 70.10 | ND | 228.00 | ND | 68.95 | ND |
| IFN-γ | 69.22 | 105.37 | 27.59 | 57.40 | 54.93 | 1.16 | 21.38 | 0.27 |
| CXCL-10 / IP-10 | 25.37 | 34.24 | 62.93 | 75.20 | 78.72 | 114.37 | 60.57 | 130.66 |
| IL-1β | 11.74 | ND | 10.02 | ND | 11.80 | ND | 9.87 | ND |
| TNF-α | 70.69 | 1283.04 | 117.03 | 523.24 | 97.20 | 85.96 | 91.20 | 64.30 |
| MCP-1 | 1571.57 | ND | 1638.62 | ND | 1633.77 | ND | 1642.39 | ND |
| IL-4 | 95.42 | ND | 208.86 | ND | 106.13 | ND | 144.73 | ND |
| IL-6 | 177.26 | 336.50 | 225.93 | 541.00 | 273.81 | 349.28 | 222.70 | 374.38 |
| IL-10 | 12.28 | ND | 23.21 | ND | 11.02 | ND | 46.18 | ND |
| IL-13 | 465.12 | 531.79 | 561.93 | 751.61 | 568.40 | 825.72 | 551.02 | 642.13 |

ND = Not Done. IFN-Interferon; TNF=tumor Necrosis Factor; CXCL-10/IP-10=Interferon Gamma-Induced Protein-10; IL=Interleukin; MCP=Monocyte Chemoattractant Protein.

*Note : (1) Anti CHIKV IgG was done in 52 symptomatic subjects(<20 years =3; 20-44 years = 19; 45-65 years = 16 and >65 years = 4) and in 16 symptomatic subjects (<20 years =1; 20-44 years = 9; 45-65 years = 4 and >65 years = 2). (2) IFN-α, IFN-β, IL-1β, MCP-1, IL-4 and IL-10 were performed in 31 symptomatic subjects (<20 years =1; 20-44 years = 15; 45-65 years = 11 and >65 years = 4). (3) IFN-γ, CXCL-10/IP-10, TNF-α, IL-16 and IL-13 were done in all subjects.

**Table S10:** **Comparison of cytokine profile of anti CHIKV seropositive (IgM and /or IgG) versus seronegative (IgM and IgG) in symptomatic cases. .** *Values expressed as Median [Standard Deviation](interquartile range) pg/ml.*

| **Cytokine**  **(pg/ml)** | Anti CHIKV Seronegative | | Anti CHIKV Seropositive | | ‘p’ |
| --- | --- | --- | --- | --- | --- |
|  | N | Value | N | Value |  |
| IFN-α | 10 | 31.02  [20.01]  (19.43 – 56.77) | 21 | 7.60  [19.46]  (5.47 – 10.05) | 0.00 |
| IFN-β | 10 | 247.48  [158.69]  (161.07 – 451.82) | 21 | 60.22  [162.34]  (44.07 – 80.10) | 0.00 |
| IFN-γ | 14 | 68.29  [514.31]  (1.41 – 139.94) | 66 | 42.59  [373.01]  (9.49 – 175.56) | 0.53 |
| CXCL-10 / IP-10 | 14 | 91.01  [282.97]  (35.66 – 524.71) | 66 | 71.33  [142.25]  (12.52 – 122.93) | 0.07 |
| IL-1β | 10 | 12.06  [1.03]  (11.24 – 13.27) | 21 | 9.66  [1.51]  (8.44 – 10.50) | 0.00 |
| TNF-α | 14 | 171.94  [302.84]  (53.81 – 623.30) | 66 | 97.20  [285.09]  (52.38 – 152.68) | 0.17 |
| MCP-1 | 10 | 1694.00  [505.06]  (1231.06 – 1741.50) | 21 | 1576.41  [607.30]  (1203.77 – 1916.55) | 0.84 |
| IL-4 | 10 | 95.42  [16.85]  (82.61 – 112.53) | 21 | 214.01  [115.05]  (127.38 – 309.66) | 0.001 |
| IL-6 | 14 | 421.17  [213.25]  (172.19 – 560.48) | 66 | 236.07  [191.73]  (641.31 – 463.99) | 0.05 |
| IL-10 | 10 | 8.61  [3.26]  (5.93 – 12.56) | 21 | 29.78  [43.77]  (14.74 – 85.56) | 0.001 |
| IL-13 | 14 | 641.53  [388.61]  (494.47 – 822.14) | 66 | 550.25  [543.52]  (436.12 – 885.44) | 0.87 |

IFN-Interferon; TNF=Tumor Necrosis Factor; CXCL-10/IP-10=Interferon Gamma-Induced protein-10; IL=Interleukin; MCP=Monocyte Chemoattractant Protein. Statistical analysis was performed using non-parametric Kruskal Wallis test.
